# Supplementary material for: Causal Relationship between Aging and Anorexia Nervosa: A White-Matter-Microstructure-Mediated Mendelian Randomization Analysis
Source: Biomedicines. 2024 Aug 16;12(8):1874. doi: 10.3390/biomedicines12081874 (PMC11351342; doi:10.3390/biomedicines12081874)
Supplement: Supplementary file 1 [file biomedicines-12-01874-s001.zip › Supplementary Figure/Figure S8/Leaveoneout plot of MR analysis for TL to ubm-b-1593.pdf]

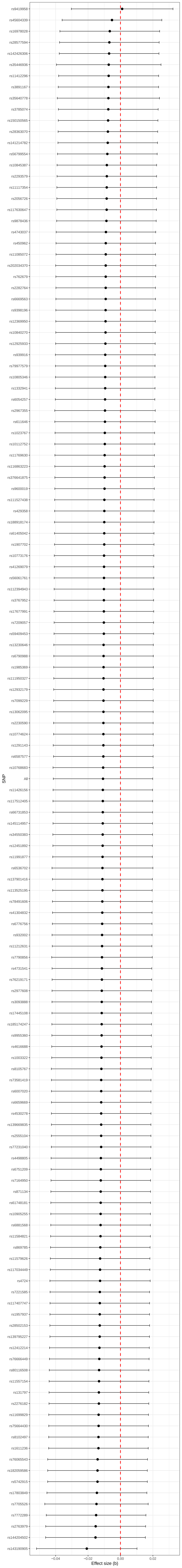
rs9419958
rs45604339
rs16978028
rs28577594
rs142426306
rs35446936
rs11412296
rs3891167
rs35640778
rs3785074
rs150150565
rs28363070
rs141214782
rs56799554
rs10845387
rs2293579
rs11117354
rs2056726
rs117630647
rs9878436
rs4743037
rs450962
rs11085072
rs202034370
rs762679
rs2282764
rs6669563
rs9398196
rs12369950
rs10840270
rs12925933
rs939916
rs79977579
rs10805346
rs1332941
rs6054257
rs2967355
rs611646
rs1023767
rs10112752
rs11769630
rs116863223
rs376641875
rs9600019
rs111527438
rs429358
rs188918174
rs61405042
rs1907702
rs10773176
rs41269079
rs56061761
rs112394943
rs3767952
rs17677991
rs7209057
rs59409453
rs13230646
rs6790988
rs1985369
rs111950327
rs12932179
rs7099229
rs13062095
rs2230590
rs10774624
rs1291143
rs6587577
rs10768683
All
rs11426156
rs117512405
rs66731853
rs145114957
rs34550383
rs12451892
rs11991877
rs6536702
rs137901416
rs113525195
rs78491606
rs41304832
rs6776756
rs932002
rs11212631
rs7790856
rs4731541
rs76219171
rs2977608
rs3093888
rs17445108
rs185174247
rs9955360
rs4616688
rs1003322
rs8105767
rs73581419
rs6007020
rs6659669
rs4530278
rs139669835
rs2555104
rs77231040
rs4498805
rs6751209
rs7164950
rs871134
rs61748181
rs10805255
rs6881568
rs11584821
rs869785
rs11579626
rs117034449
rs4724
rs7221585
rs117407747
rs1957937
rs28502153
rs139795227
rs12412214
rs76666449
rs80116508
rs11557154
rs131797
rs2276182
rs11699829
rs75664430
rs8102497
rs1631236
rs76065543
rs182059586
rs5742915
rs17803849
rs7705526
rs7772289
rs2763979
rs144204502
rs143190905
SNP
-0.04
-0.02
0.00
0.02
Effect size (b)
